# Ultrahigh Piezoelectric Strains in PbZr_1−x_Ti_x_O_3_ Single Crystals with Controlled Ti Content Close to the Tricritical Point

**DOI:** 10.3390/ma15196708

**Published:** 2022-09-27

**Authors:** Iwona Lazar, Roger William Whatmore, Andrzej Majchrowski, Anthony Mike Glazer, Dariusz Kajewski, Janusz Koperski, Andrzej Soszyński, Julita Piecha, Barbara Loska, Krystian Roleder

**Affiliations:** 1 Institute of Physics, University of Silesia, ul. 75 Pułku Piechoty 1, 41-500 Chorzów, Poland; 2 Department of Materials, Faculty of Engineering, Imperial College London, London SW7 2AZ, UK; 3 Institute of Applied Physics, Military University of Technology, ul. Kaliskiego 2, 00-908 Warsaw, Poland; 4 Clarendon Laboratory, University of Oxford, Oxford OX1 3PU, UK; 5 Department of Physics, University of Warwick, Coventry CV4 7AL, UK

**Keywords:** ABO_3_ perovskites, ferroelectric and antiferroelectric phase transitions, PZT single crystals, piezoelectricity, tricritical point, birefringence, polar regions

## Abstract

Intensive investigations of PbZr_1-x_Ti_x_O_3_ (PZT) materials with the ABO_3_ perovskite structure are connected with their extraordinary piezoelectric properties. Especially well known are PZT ceramics at the Morphotropic Phase Boundary (MPB), with x~0.48, whose applications are the most numerous among ferroelectrics. These piezoelectric properties are often obtained by doping with various ions at the B sites. Interestingly, we have found similar properties for undoped PZT single crystals with low Ti content, for which we have confirmed the existence of the tricritical point near x~0.06. For a PbZr_0.95 ± 0.01_Ti_0.05__∓ 0.01_O_3_ crystal, we describe the ultrahigh strain, dielectric, optical and piezoelectric properties. We interpret the ultrahigh strain observed in the region of the antiferroelectric–ferroelectric transition as an inverse piezoelectric effect generated by the coexistence of domains of different symmetries. The complex domain coexistence was confirmed by determining optical indicatrix orientations in domains. The piezoelectric coefficient in this region reached an extremely high value of 5000 pm/V. We also verified that the properties of the PZT single crystals from the region near the tricritical point are incredibly susceptible to a slight deviation in the Ti content.

## 1. Introduction

The first phase diagram for PbZr_x_Ti_1-x_O_3_ (PZT) solid solutions, which was proposed by Sawaguchi in 1953 [1], was derived only from measurements on ceramics. However, even now, nearly seven decades since the system’s discovery, only a few papers have reported the properties of PZT single crystals [2,3,4,5,6,7,8,9], mainly because of the difficulties in the technology involved in their growth [10]. Ceramics are much easier to make, so most papers on PZT have been on ceramics, especially at compositions around the MPB region, PbZr_0.52_Ti_0.48_O_3_ [11], where there is a boundary between ferroelectric rhombohedral (F_R_) and ferroelectric tetragonal (F_T_) phases, and where the large piezoelectric effect was found. Because of this strong piezoelectricity, ceramics with compositions in this region have found many practical applications. The discovery of a monoclinic phase in the MPB region by Noheda [12] caused a considerable re-awakening of interest in the fundamentals of this system and led to a re-examination of Sawaguchi’s phase diagram [13,14]. Compositions close to the Ti-end of the PZT-system have also been of interest for high-frequency piezoelectric and pyroelectric applications [15,16]. Compositions at the low Ti composition range of the phase diagram, close to lead zirconate, are mainly of interest for pyroelectric applications [17,18], and this potential was underlined by the discovery in 2006 of giant electrocaloric effects around the Curie T_C_ temperature in PbZr_0.95_Ti_0.05_O_3_ thin films [19], leading to an explosion of interest in the possibility of using the electrocaloric effect for solid-state cooling. One curious observation was made by Whatmore et al. [20], who found a tricritical point in the F_R_ to cubic (P_C_) phase transition for compositions with *x*~0.06, and also reported significant differences between the phase transition temperatures of single crystals and ceramics at this point. Particularly striking was a peak in the T_C_ vs. composition graph at the tricritical point in the single-crystal measurements, which was absent for the ceramics. Subsequently, other tricritical points have been located in the PZT system at x = 0.38 [21] and x = 0.43 [22].

Recently, we have succeeded in growing good quality low-Ti content PZT single crystals by the Top-Seeded Solution Growth (TSSG) technique. Based on their optical and dielectric properties, we have confirmed the tricritical point in these crystals. More importantly, we have found that in crystals of compositions near this point, piezoelectric strains are higher than for MPB ceramics [23]. This is true also for PZT ceramics with dopants at the B site [24,25,26], perovskite lead-free ceramics [27,28] (<1000 pm/V) or even the best relaxor single crystals such as PZN-PT or PMN-PT (~2500 pm/V) [29,30]. The ultrahigh strain, observed in the region of the antiferroelectric (AFE)–ferroelectric (FE) transition, is interpreted as an inverse piezoelectric effect generated by the coexistence of domains of different symmetries. We also verified that the properties of the PZT single crystals from the region of the tricritical point are very susceptible to slight deviations in the Ti content.

## 2. Materials and Methods

PZT single crystals, because of their incongruent melting, have to be grown from a flux to lower the crystallisation temperature below the temperature of the peritectic phase transition. Pb_3_O_4_ (99.8%, ABCR), ZrO_2_ (99.99%, Merck), and TiO_2_ (99.99%, Merck) were used as precursors in PZT synthesis. The crystallising solution contained 2.4 mol% of PZT. The commonly used solvent is a mixture of PbO and B_2_O_3_ in 4:1 molar ratio. This provides a broad temperature range of crystallisation (1200–900 °C) and does not introduce impurities into the growing PZT single crystals because boron ions are too small to remain in the crystal structure. The B_2_O_3_ addition also influences the viscosity of the flux and diminishes the losses of PbO caused by evaporation. We have modified the composition of the flux by using Pb_3_O_4_ instead of PbO as a solvent and source of Pb^2+^ ions. Owing to its oxidising properties (presence of PbO_2_), we managed to improve the optical quality of the growing PZT single crystals, and the grey coloration of PbO-grown PZT single crystals was eliminated. Spontaneous crystallisation and Top Seeded Solution Growth (TSSG) were used in our PZT crystal growth experiments. The latter method is commonly used in the crystallisation of incongruently melting materials as this makes it possible to influence numerous parameters of the crystallisation process, such as seed orientation, mass transport in the melt, as well as the thickness of layer boundaries, by changing the rotation rate. It is also possible to reduce the temperature gradient in the vicinity of the growing crystal, thus avoiding the formation of supercooling conditions, which can lead to crystals containing inclusions. Despite its limitations because of lack of control of these parameters, the former method gives much better quality PZT single crystals. One of the possible reasons for this is probably because there are more stable conditions of mass transport near the bottom of the Pt crucible, where spontaneous crystallisation occurs. The other factor making TSSG conditions less favorable may be some changes in composition of the PbO-B_2_O_3_ flux near the surface of the melt coming from increasing content of B_2_O_3_ by PbO evaporation and leading to the formation of some additional compounds, thus worsening the conditions by influencing the composition of the boundary layer. One of such compounds is PbZr(BO_3_)_2_, crystallisation of which was observed during attempts of PbZrO_3_ crystallisation from PbO-B_2_O_3_ melts enriched in B_2_O_3_. In the case of PZT crystallisation, the whole range of mixed PbZr_1-x_Ti_x_O_3_ compounds may be formed. Energy-dispersive X-ray spectroscopy (EDS) was used to investigate the chemical composition of the crystal. With this technique, the determination of the Ti content was possible with an error of 1%.

Dielectric measurements were conducted using a standard capacitance method using an Agilent 4192A Impedance Analyzer at a frequency range 1 kHz-1 MHz and electric field strength not exceeding 0.1 kV/cm. The temperature was controlled with a precision of the order of 0.05 K, and measurements were performed at a temperature rate of 0.2 K/min. For a defined angular frequency ω of the electric field, the real ε′ and imaginary ε″ parts of the permittivity were calculated from the relation ε′ = C/C_0_ and ε″ = G/ωC_0_. C and G are respectively the capacity and conductivity of the sample, and C_0_ is a vacuum condenser with sizes equal to the crystal surface, its thickness, and the free space permittivity ε_0_.

Birefringence measurements were made employing an Oxford Cryosystems Metripol Birefringence Imaging System (Metripol, Oxford, UK). Details of the technique have been described elsewhere [31,32,33]. The sample was heated in a high-precision Linkam THMS600 (Surrey, UK) temperature stage. The hot stage was capable of maintaining a constant temperature to within 0.1 K, and the temperature ramps were performed at rates of 0.2 K/min. We determined the values of birefringence ∆n in the following way. The change in the optical retardation Γ = (Δn)⋅k for specimens of thickness k of the order of hundreds of micrometers allows the variation of Γ with temperature to be determined. The maxima of the periodic output signal from the rotating-analyser apparatus occur provided $\Gamma \;=\frac{\left(2N\;+1\right)\lambda }{4}\;$, where N is an integer and λ is the wavelength of the light. Hence, the birefringence was calculated by the formula $∆n\;=\frac{\left(2N\;+1\right)\lambda }{4k}\;$.

The field induced strain was measured and recorded using a method described earlier [34] and summarised in greater detail in the Supplement Materials S1. The method uses a capacitive sensor to determine the field induced strains in the same direction as applying an alternating electric E field with a frequency f = 70 Hz. This is transferred via a quartz rod with one end placed on the sample surface and the other connected to the capacitor sensor plate. A lock-in amplifier is used to determine the maximum strain in synchrony with the applied field. The deformation signal appearing at the same frequency f as the applied E field is assigned as the “piezoelectric” strain, which will be referred to throughout this text as “effective” piezoelectric coefficient $d_{33}^{e}$. Strains appearing at the doubled frequency 2f = 140 Hz are assigned as “electrostrictive” and being produced by a reorientation of domains in the alternating electric field.

## 3. Results

### 3.1. Tricritical Point

This section describes the optical and dielectric properties of PbZr_1-x_Ti_x_O_3_ single crystals with Ti content 0 ≤ x ≤ 0.13 as a function of temperature. The as-grown PZT crystals used in experiments were oriented in the [1] pseudocubic direction. From these measurements, we can confirm the existence of the tricritical point at a composition of around x = 0.06. The EDS accuracy in the determination of chemical composition was of the order of x = 0.01. Information on EDS studies of selected crystals is in Supplement Materials S2.

**Figure 1:**
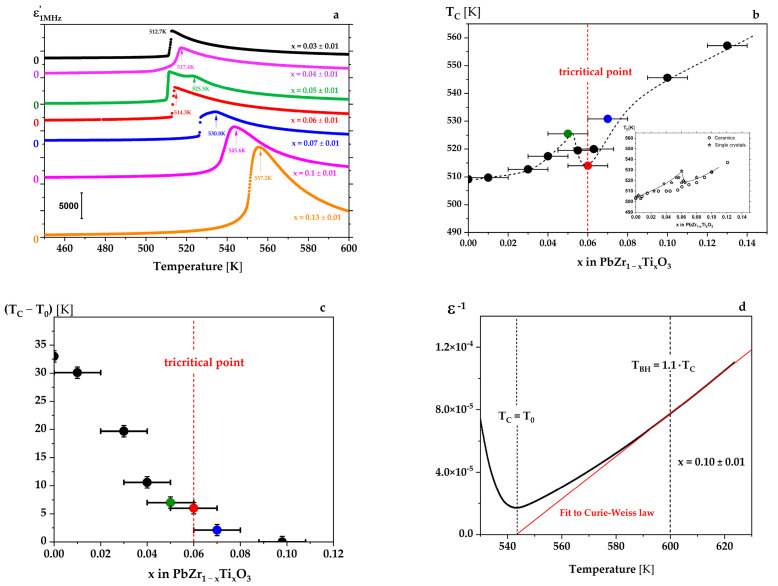
(**a**) Permittivity as a function of temperature on heating for PZT single crystals with different Ti contents. On cooling, the dependences are very similar, undergoing 1.5 K thermal hysteresis only. Note the complex variation of ε′ (T) near the transition point for compositions with x~0.06 ± 0.01. (**b**) The compositional variation of T_C_ is defined as that at which the domain structure disappears during the transition from the rhombohedral to cubic phase (see arrows in (**a**)). The T_C_ (x) dependence (dashed line is a guide for the eyes) is similar to that shown in the inset. The inset represents changes of T_C_ (x) for ceramics and single crystals, with a local extremum at T_C_ for the single crystal at the tricritical point for x = 0.06 ± 0.01 observed by Whatmore et al. (redrawn from [20]) and denoted by the red dashed line in (**b**). Points for x = 0 and 0.01were taken from [35,36]. (**c**) The compositional variation of the difference (T_C_ − T_0_) in PbZr_1-x_Ti_x_O_3_ single crystals. (T_C_ − T_0_) diminishes towards zero at around *x* = 0.10, as would be expected for a second-order phase transition. (**d**) The Curie-Weiss law obeyed for crystals with *x* = 0.10, for which T_c_ = T_0_ is characteristic of a continuous (second order) phase transition.

The temperature variations of the real part of the permittivity ε′ in PZT single crystals are shown in Figure 1a. T_C_ has been defined as the point at which the domain structure disappears. We investigated the dependence ε′(T) for many crystals with the Ti content close to x = 0.06. The Figure 1a–c contain the most representative runs and prove that the properties of the PZT single crystals are very susceptible to a slight deviation in the Ti content near the tricritical point.

The T_C_(x) dependence is similar to that found in [20], with the appearance of a local maximum near x = 0.06 ± 0.01 (Figure 1b). The dependence of ε′ (T) for x = 0.05 ± 0.01 reveals an additional anomaly below T_C_ (Figure 1a), and as we discuss further in the paper, this corresponds to a change in the mobility of the ferroelectric/ferroelastic domains. However, one cannot exclude the possibility that the region between this additional anomaly and T_C_ corresponds to the existence of another phase. This possibility has recently been reported [37], where it was speculated that it might be connected with the disorder in the array of octahedral tilts (see Figure 7 and temperature T_IT_ in that paper). Such an additional anomaly has also been observed near 453 K for a single crystal with *x* = 0.13 [38]. To check if a crossover from first-order (discontinuous) to second-order (continuous) behaviour at the FE to P phase transition takes place in the PZT system at around x = 0.06 ± 0.01, as reported previously [20], the compositional variation of (T_C_ − T_0_) for the single crystals is plotted in Figure 1c. The T_C_ value corresponds to the disappearance of the domain structure, and the T_0_ value was obtained from the fit to the Curie–Weiss law ε′ (T) = C/(T − T_0_) + ε_∞_, where ε_∞_ is permittivity equal to the square of the refractive index *n^2^*, i.e., ε_∞_ = n^2^ = 6. This law is fulfilled in a temperature region above T_C_, over the temperature T_BH_ = 1.1T_C_ found by Bussmann–Holder et al. [39]. Between the T_C_ and T_BH_, specific phonon instabilities, caused by the interaction of the optical and acoustic soft modes, break the locally cubic symmetry and lead to the appearance of polar regions above T_C_. That is why in the range T_C_ < T < T_BH_ there is a deviation of the ε′^−1^(T) run from a linear dependence related to a precursor effect connected with the coexistence of polar regions in the paraelectric matrix, as discussed in [39] and experimentally proved in pure BaTiO_3_ and PbZrO_3_ [40,41]. According to this procedure, the difference (T_C_ − T_0_) gradually tends toward zero, and finally, for a single crystal with *x* = 0.10, it is not detectable. Extrapolating the value of (T_C_ − T_0_) to zero predicts the tricritical point near x = 0.06 ± 0.01, as reported earlier [20,42,43].

**Figure 2:**
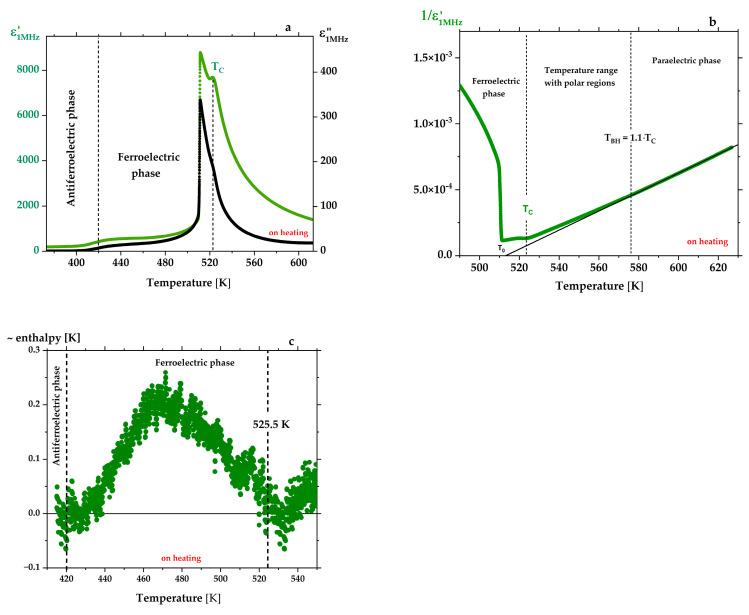
(**a**) Temperature dependence of the real ε′ (in green) and imaginary ε″ (in black) part of the permittivity for PbZr_0.95 ± 0.01_Ti_0.05__∓ 0.01_O_3_ crystal. (**b**) As in Figure 1d, the 1/ε′(T) function obeys the Curie–Weiss law only above the temperature T_BH_, which limits polar regions existing above T_C_ and below T_BH_. (**c**) During the discontinuous phase transition, a change in the enthalpy takes place, and its changes can be recorded through the temperature changes (measured by the thermocouple placed close to the crystal) of 0.2 K/min with simultaneous measurement of ε′(T). These enthalpy changes start near the AFE-FE transition and disappear around 525 K, i.e., close to the smaller maximum seen in ε′(T) run in (**a**), and justify the placement of T_C_ at this point.

The dielectric response for PbZr_0.95 ± 0.01_Ti_0.05__∓__0.01_O_3_ crystal with a thickness of 70 µm in Figure 2a reveals three anomalies. The first transition ~420 K from the AFE to the FE phase, the second at 511 K corresponds to the maximum permittivity and dielectric losses, and the third one at 525.5 K (T_C_) is due to the FE-P phase transition. We have additionally checked that the transition points are related to changes in crystal temperature due to enthalpy at AFE-FE and FE-P (T_C_) transitions (see Figure 2c).

#### Optical Measurements

**Figure 3:**
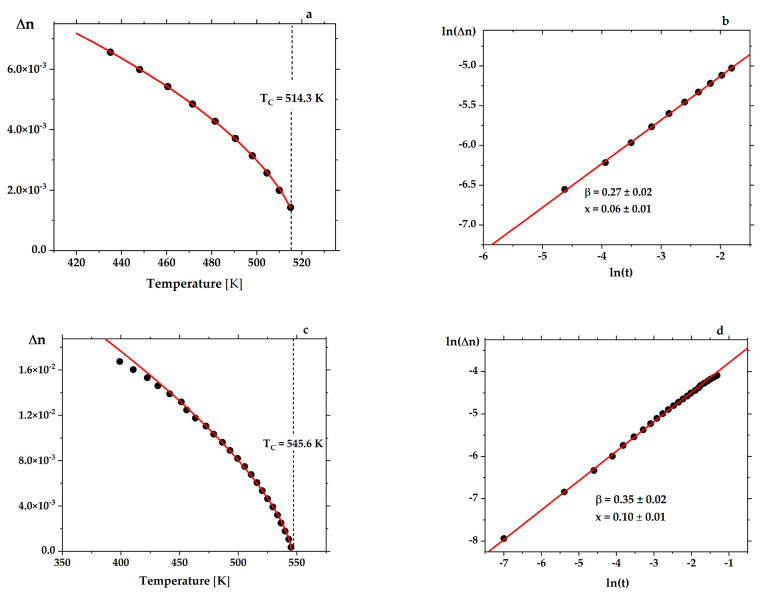
Temperature dependence of birefringence ∆n for 500 µm thick PbZr_0.96 ± 0.01_Ti_0.04__∓ 0.01_O_3_ (**a**) and 400 µm thick PbZr_0.90_Ti_0.10_O_3_ (**c**); logarithmic dependences of the birefringence ∆n versus reduced temperature t allow for determining the exponent β (**b**,**d**). The red curves fit the relation (3), and the value of T_C_ was found from the best linear least-squares fit to the logarithmic dependence. The calculated values of the Curie point correspond closely to those determined from the dielectric measurements for the same samples (see Figure 1). For a crystal with x = 0.06 ± 0.01, the β value equal to 0.27 ± 0.02 confirms its tricritical behavior (**b**). The birefringence curve for PbZr_0.90_Ti_0.10_O_3_ crystal corresponds to that for a second-order phase transition, as previously reported by Glazer et al. [44] (**d**). The value β = 0.35 ± 0.02 obtained for this crystal is almost the same (β = 0.33) as that found by Clarke and Glazer [10].

The PZT single crystals obtained were highly transparent for birefringence measurements over a broad temperature range. Below the transition point T_C_, for crystals for which continuous transitions were expected, such measurements enabled exponents to be computed according to the relation:

∆n(T) ~ (Tc − T)^2β^ (1)

for which exponent β = 0.5 is expected for a continuous phase transition (second order), and β = 0.25 at a tricritical point. In this equation, ∆n is the linear birefringence and T_C_ the Curie temperature. The relation (1) can be written in the following form:

∆n(t) = const · (t^2β^) (2)

where t is the reduced temperature given by the ratio (T_C_ − T)/T_C_. Having measured ∆n as a function of temperature, the exponent β can be determined from the linear relation (Figure 3):

ln(∆n) = ln(const) + 2β ln(t)(3)

### 3.2. Ultrahigh Piezoelectric Strains in PbZr_0.95 ± 0.01_Ti_0.05__∓ 0.01_O_3_ Single Crystals

**Figure 4:**
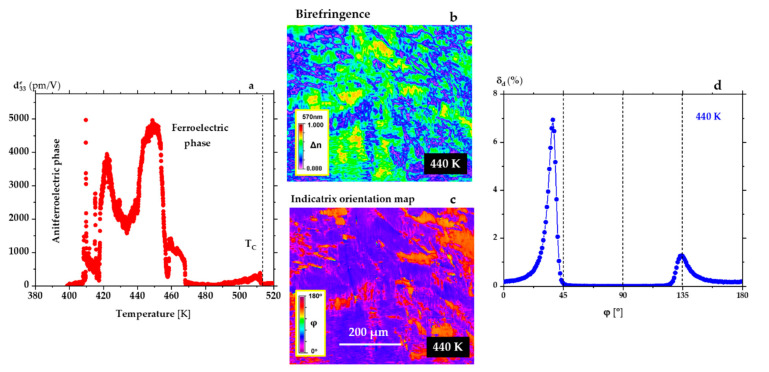
(**a**) Giant piezoelectric effect recorded on heating, in the temperature range between the AFE-FE and FE-P phase transitions in a PbZr_0.95 ± 0.01_Ti_0.05__∓ 0.01_O_3_ single crystal (green curve in Figure 1a and Figure 2a). Such a violent jump at the AFE-FE is connected with the violent appearance of complex domain structure under the quartz rod which transforms strain to the capacitance sensor. The signal is taken from dozens of square micrometres only. For detailed information about the temperature changes of domains, see the movie from Supplement Materials S3. Figures (**b**,**c**) represent birefringence ∆n and optical indicatrix orientation maps at 440 K in the temperature range where the ultrahigh piezoelectric coefficient was observed. The inserts on the maps show colours corresponding to the values of ∆n and ϕ. (**d**) Example of domain populations δ_d_ at 440 K determined from the map in (**c**) (c axis is perpendicular to the crystal surface).

For the single crystal PbZr_0.95 ± 0.01_Ti_0.05__∓ 0.01_O_3_ (green curve in Figure 1a and Figure 2a), a diffuse phase transition was observed from the AFE phase to the FE phase. The ε′(T) increases continuously over this transition range from 420 K to 470 K (see Figure 2a). Crossing this phase transition, the strain was measured as a function of temperature along the electric field direction as described in the Methods section. Measurement of η_3_(T) allowed us to determine the dependence of $d_{33}^{e}\left(T\right)$, and to register a very high value of the effective piezoelectric modulus $d_{33}^{e}\;$= 5000 pm/V (Figure 4a). As shown in Figure 4b,c, in the range of the enhanced piezoelectric effect, the domain population contains rhombohedral and monoclinic (φ > 120° and φ < 45°) domains [45]. The Metripol system allows measuring the optical indicatrix orientation between the 0° and 180°. So, the orientations in the case of rhombohedral symmetry are possible at 45° and 135°. Only the second case is realised in Figure 4d, which presents indicatrix orientation at 440 K. It is worth noting that there are also minor concentrations of domains of the tetragonal symmetry (non-zero orientations for φ = 0° and 90°).

**Figure 5:**
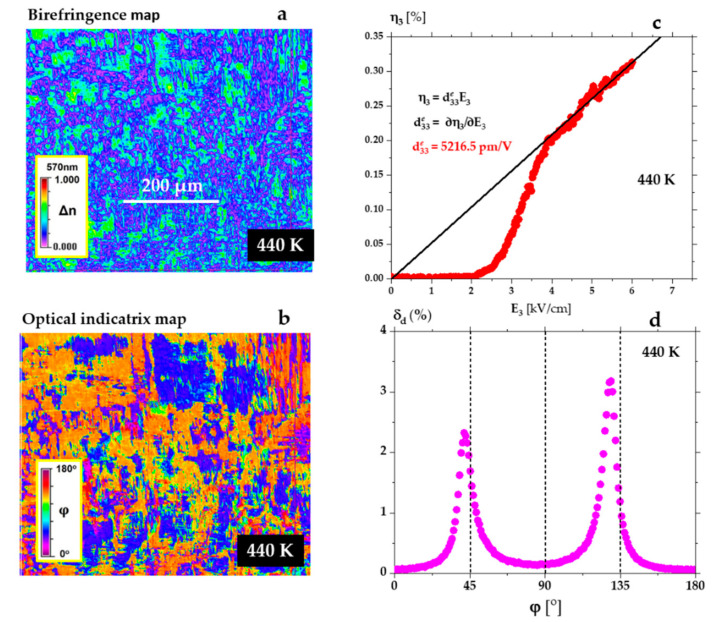
Figures (**a**,**b**) represent birefringence ∆n and optical indicatrix ϕ maps at 440 K at which the ultrahigh piezoelectric coefficient was observed. (**c**) Giant piezoelectric effect recorded at 440 K for increasing electric field amplitude, in a PbZr_0.95 ± 0.01_Ti_0.05__∓ 0.01_O_3_ single crystal. (**d**) Example of domain populations δ_d_ at 440 K after long-time action of the AC field, determined from the map in (**b**). Comparing with Figure 4d shows additional monoclinic orientations in the range of 45° and 135°.

In a temperature range similar to that in Figure 4, we have discovered that after long-time action of the AC field on this single crystal, there is a higher field induced strain. The dependence of strain η_3_ in this single crystal at 440 K as a function of the electric field E is shown in Figure 5. This strain η_3_ was up to 0.3% (Figure 5c), a figure previously observed only in relaxor single crystals [46]. Figure 5b,d show that the domain population accompanying the huge piezoelectric effect is represented by any domain orientation but with overwhelming populations of monoclinic symmetry. This is because most of the populations have orientations below 45° and 135° orientation, characteristic of monoclinic symmetry [45].

## 4. Discussion

**Figure 6:**
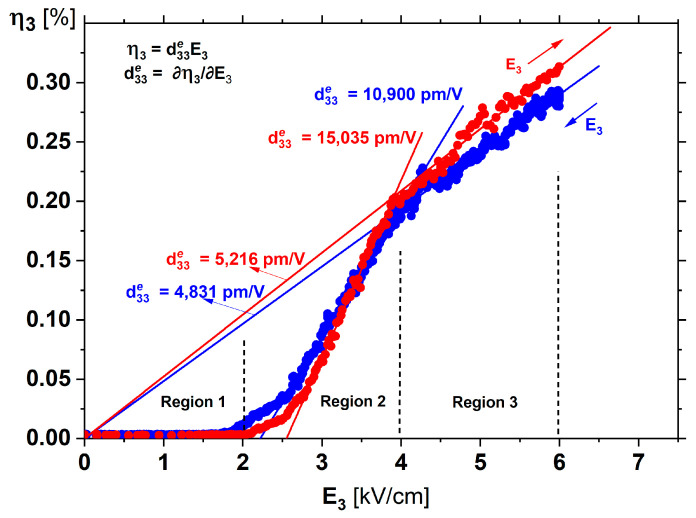
Giant effective piezoelectric effect recorded at 440 K for increasing (red) and decreasing (blue) AC electric field in a PbZr_0.95±0.01_Ti_0.05__∓0.01_O_3_ single crystal. For a description of the individual regions, see text.

The exceptionally high strain-field behaviour at 440 K, presented in Figure 5c, can be divided into three regions (Figure 6). A weak piezoelectric effect is observed in Region 1, below an AC field of 2 kV/cm amplitude. At the beginning of Region 2, from ~2 to 2.5 kV/cm, there is a non-linear dependence of strain on the field. But there is a substantially linear section between ~3 and 4 kV/cm, for which the $d_{33}^{e}$ takes on the value of ~15,000 pm/V. This is the highest value measured in ABO_3_ perovskites and is connected with the change in the domain population with monoclinic symmetry caused by the electric field being of sufficient value to induce such changes. Such a dynamic change can also be related to the movement of the domain walls, which additionally increases the $d_{33}^{e}$ value [47]. The increasing number of monoclinic domains allows, at the same time, ease of orientation of the polarisation together with a rapid increase in strain. Enhancement of piezoelectric properties because of such an extrinsic effect was recently observed in [48]. Region 3 is for fields higher than ~4 kV/cm, with the most striking η_3_(E_3_) dependence. The linear dependence is characteristic of a piezoelectric relation $d_{33}=\frac{\partial \eta_{3}}{\partial E_{3}}$ (note that the linear portion of this curve extrapolates to zero at zero fields). This gives a value for the piezoelectric coefficient in this region around 5000 pm/V. For these fields, it was checked that the electrostrictive strain—coming from the reorientation of the domains at a frequency double that of the applied AC field—was overwhelmed by the piezoelectric response. Fourier transformation of the measured strain signal showed that the amplitude of the piezoelectric component of the strain was more than 25 times greater than the electrostrictive component.

The temperature range of existence of the giant piezoelectricity correlates with very fine domains with the dominant population of monoclinic symmetry. In the Supplement Material, the film shows that inside the antiferroelectric phase at 80 °C/353 K, the crystal is in a single-domain state. This is represented by homogenous colours changing with increasing temperature (a feature of the Metripol technique). When the AFE—FE transition starts at 147 °C / 420 K, this single-domain state decomposes into a multidomain state, represented by numerous different colours. Interestingly, the temperature range of the existence of the giant piezoelectricity correlates with this multidomain state with a dominant population of domains with monoclinic symmetry. Figure 5b,d show that the axis of the optical indicatrix inside the domains is oriented in entirely different directions to the crystal edges of [100] orientation. In the range of electric field greater than 4 kV/cm (Region 3), the high value of the $d_{33}^{e}$ can be explained in the following way. Of all possible symmetries considered so far for PZT (see Table 1 in [45]), the piezoelectric matrices for monoclinic phases contain many non-zero piezoelectric coefficients, among them those describing shear strains, as d_15_. A scenario for this Region 3 is the following. A strong electric field causes a change in the direction of the polarisation vector inside the monoclinic phases in the direction of the pseudocubic c-axis, and the crystal effectively reveals a giant piezoelectric response. The temperature range of existence of the giant piezoelectricity correlates with very fine domains in the dominant population with monoclinic symmetry (Figure 5d).

## 5. Conclusions

Through dielectric and optical measurements of high-quality single crystals, we have confirmed the existence of the tricritical point at the ferroelectric–paraelectric (FE-P) phase transition in the Zr-rich PZT system. The FE-P transition in crystal PbZr_0.94 ± 0.01_Ti_0.06__∓ 0.01_O_3_ corresponds to a crossover between first and second-order transitions, and in PbZr_0.90 ± 0.01_Ti_0.10__∓ 0.01_O_3_ crystal a second-order transition was observed. Furthermore, we have shown that for a PbZr_0.95 ± 0.01_Ti_0.05__∓ 0.01_O_3_ single crystal, in the region of the tricritical point, repeatable giant reversible effective piezoelectric effects occur, with coefficients of up to around 5000 pm/V. These are much greater than any others reported, even in relaxor single crystals, and we believe they are an essential indicator for future research directions in piezoelectric materials. It is fascinating that this effect has been observed in materials of the same composition as that in which the giant electrocaloric effect was discovered in thin films. As an easy E field reorientation of the ferroelectric polarisation is required for both effects, it is likely that the results are closely connected. We suggest that appropriate doping of the crystals to reduce the relevant transition temperatures (e.g., by use of La, which is well known to reduce T_C_ in PZT) might bring the effect into a more technologically relevant temperature range.

Moreover, we have discovered that an enhancement of the piezoelectric properties up to 15,000 pm/V is possible, provided that there are domains that can easily be transformed to lower symmetries under electric field action. Such a transformation is possible for domains that evolve during the antiferroelectric–ferroelectric transition and quickly transform into monoclinic domains (Figure 5d). Such a change in the population of the domains, including those with monoclinic symmetries M_A_, M_B_, and M_C_, leads to the giant piezoelectric effect, such as we observed in Region 3 in Figure 6.

It must also be stressed that PbZr_1-x_Ti_x_O_3_ single crystals from the region of the tricritical point at x ~ 0.06 are hugely sensitive to Ti content. Even small changes in Ti concentration lead to drastic changes in the shapes of the temperature dependence of permittivity near the Tc point (Figure 1a).

## Data Availability

Data is contained within the article or supplementary material.

## Supplementary Materials

The following supporting information can be downloaded at: https://www.mdpi.com/article/10.3390/ma15196708/s1, Figures S1-1 and S1-2: Supplement Materials S1; Figures S2-1 and S2-2, Information on EDS studies: Supplement Materials S2; Videos: Supplement Materials S3, Please take it from the link https://1drv.ms/p/s!Ajbi8KG9cs8Ijj2mm1smf8nD2C_Z?e=yWUlPu.
