# Supplementary material for: Ultrahigh Piezoelectric Strains in PbZr1−xTixO3 Single Crystals with Controlled Ti Content Close to the Tricritical Point
Source: Materials (Basel). 2022 Sep 27;15(19):6708. doi: 10.3390/ma15196708 (PMC9572843; doi:10.3390/ma15196708)
Supplement: Supplementary file 1 [file materials-15-06708-s001.zip › materials-1933521-supplementary S1.pdf]

Supplement Materials S1

Below is described the method of the quasi-static strain measurements.

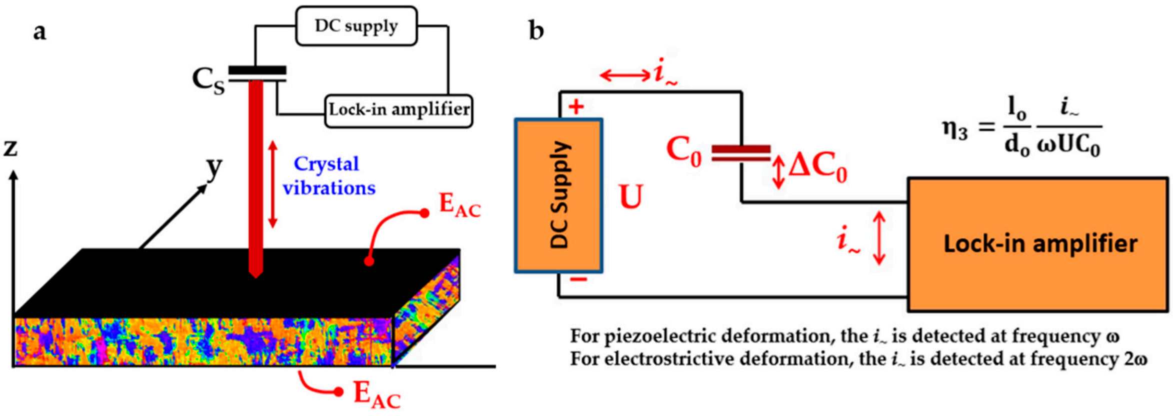

Figure S1-1. a) Detection of strain as a function of electric field E_{AC} of frequency f=70 Hz and strength up to 6 kV/cm. C_S is the sensor capacity to which a DC voltage U=90 V was applied. A lock-in amplifier was used to detect the alternating current, which was proportional to the sample strain η_3 and induced by changes ΔC_S of the sensor capacity C_S. **b)** Scheme of the experimental setup for quasi-static measurements of strain. Current $i_\sim$ is measured by a lock-in amplifier at the same frequency as the E_{AC} field due to changes in the C_S. The t_0 is the sample thickness, and l_0 is the distance between the plates of the C_S sensor. Details of the measurement method can be found in [1].

Strain η_3 was induced by an alternating electric field E_{AC} of frequency 70 Hz and amplitude up to 6 kV/cm applied to the sample and transferred via a quartz rod with one end placed on the sample surface (Figure **S**1-1a). The other end was connected to the plate of a capacitor sensor C_S. The frequency of 70 Hz was chosen to minimise external noise and experimental errors. The surface area of the end of the quartz rod touching the crystal was of the order of tens of μm^2. The field amplitude increase was automatically controlled with a rate of 0.02 kV/cm per minute. Field-induced deformation was measured synchronously with the applied voltage frequency f, causing changes ΔC_S of the sensor capacity C_S, according to the time-dependent function $C_S(t) = C_{S0}+\Delta C_S \sin(2\pi ft)$. The initial value C_{S0} was adjustable depending on the magnitude of the strain measured, and ΔC_S was determined by recording the current using a lock-in amplifier. The maximum strain η_3 value was calculated as the maximal value of current $i_\sim$ registered by the lock-in amplifier at the same frequency as the AC field and for electric field E_{AC} amplitude. Measurements for increasing the AC field mean that each experimental point, as in pink in Figure S2, was an average of 25 values of $i_\sim$ corresponding to a continuously acting E_{AC} field. The electric field strength E_{AC} increased and decreased at a constant rate of 0.2 kV/minute, i.e., the run $\eta_3(E_{AC})$, for increasing and decreasing field, lasted 40 minutes.

The strain η_3 is produced by polarisation vector movement in the monoclinic unit cells, which are differently oriented to the AC field direction (see Figures 4 and 5 in the main text). Reorientation of the domains does not occur here because the electrostrictive strain connected with domain reorientation appears at doubled frequency 2f and could be neglected.

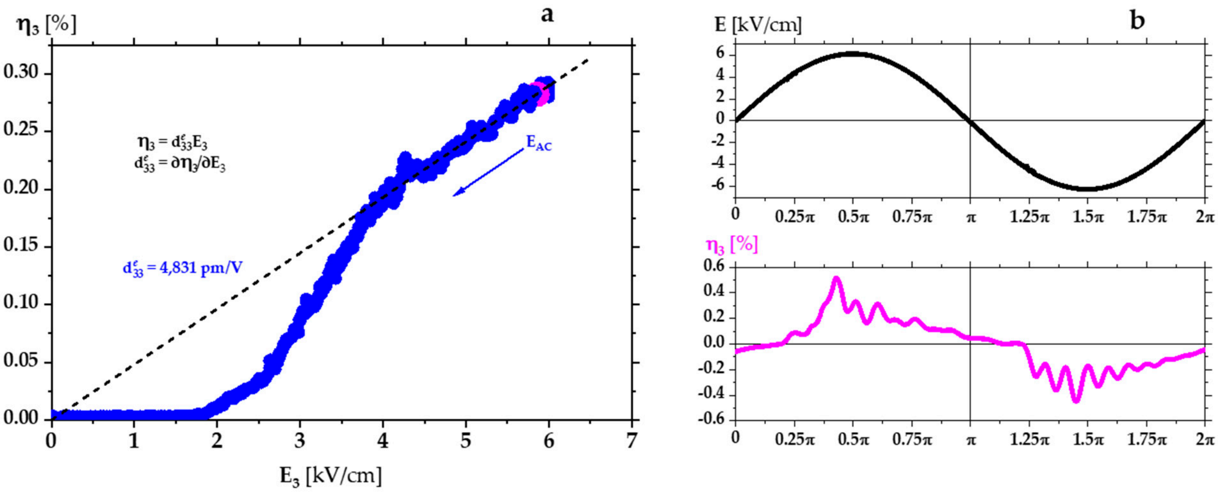

Figure S1-2. a) Strain η_3 as a function of decreasing E_{AC} field, measured by lock-in amplifier at frequency 70 Hz. The pink experimental point represents the effective strain of the $\eta_3(\omega t)$ run shown in pink colour in the Figure on the right. **b)** Total $\eta_3(\omega t)$ and $E_{AC}(\omega t)$ run in one period of E_{AC} of 6 kV/cm strength registered by an oscilloscope at the same time. The runs of a similar shape were observed for representative experimental points denoted in cyan colour in Fig. **S2**a.

It is seen that the strain changes sign according to the E_{AC} sign, as is expected for the inverse piezoelectric effect. Irregularities visible in $\eta_3(\omega t)$ run in Figure **S1-**2b are connected with the polarisation rotations taking place in the individual monoclinic domains (see Figure 5 in the main text), but these are not complete domain reorientations. If it had been so, we would have registered an electrostrictive signal at a double frequency, as one observes when a typical ferroelectric P(E) hysteresis loop occurs, but it was negligible. The source of these irregularities is the non-uniformity of the rotation of polarisations in individual domains, differently oriented to the direction of the acting field. One could say that these irregularities are incomplete *butterfly* loops for individual domains. The fact that polarisation rotations do not occur simultaneously in all domains may be due to their unequal sizes and the presence of domain walls, in which extended defects like dislocations causes domain pinning. Moreover, the observed irregularities contain yet another interesting phenomenon. While the contribution of polarisation switching in the domains to the total strain is comprehensible, the instantaneous oscillation of the strain when E_{AC} field strength increases is somewhat unexpected. This indicates a strain relaxation process, which nature requires further study.

Regardless of these uncorrelated polarisation rotations, the crystal's effective deformation satisfies the linear dependence on the increasing E_{AC} field and behaves like a colossal inverse piezoelectric effect.

[1] Roleder, K., *Measurement of the high-temperature electrostrictive properties of ferroelectrics*. Journal of Physics E: Scientific Instruments, 1983. 16(12): p. 1157.
