# Supplementary material for: Ultrahigh Piezoelectric Strains in PbZr1−xTixO3 Single Crystals with Controlled Ti Content Close to the Tricritical Point
Source: Materials (Basel). 2022 Sep 27;15(19):6708. doi: 10.3390/ma15196708 (PMC9572843; doi:10.3390/ma15196708)
Supplement: Supplementary file 1 [file materials-15-06708-s001.zip › materials-1933521-supplementary S2.pdf]

Supplement Materials S2

Below are two Figures that contain representative data of the EDS studies. Distributions of the Pb, Ti and Zr atoms were examined using the scanning electron microscopy (SEM) technique by the JSM-5410 Oxford Instruments device, supplied with an energy dispersion x-ray spectrometer (EDS). The set was also equipped with an energy dispersion x-ray spectrometer with Si(Li) x-ray detector. The vacuum in the test chamber was 10^{-4}-10^{-5} Pa. The chemical composition was determined using ISIS-300 SEMQuant software.

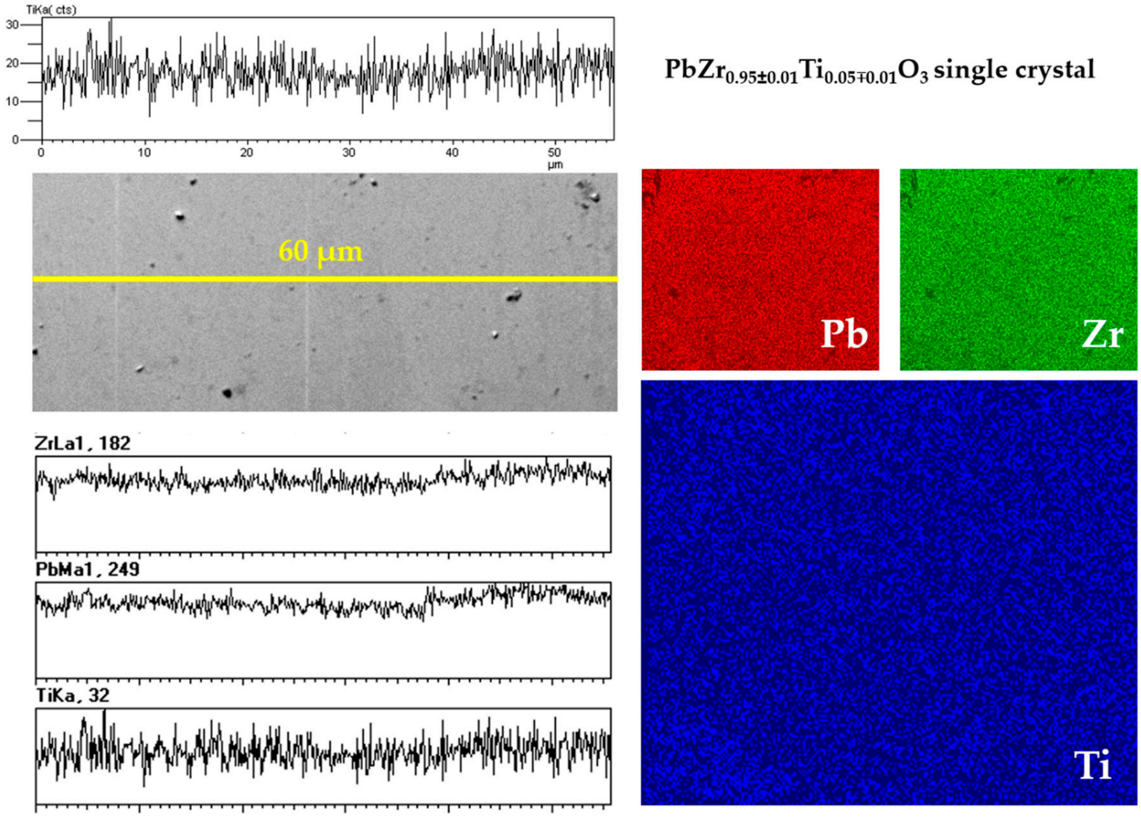

Figure S2-1. SEM analysis for as-grown $PbZr_{0.96\pm0.01}Ti_{0.04\mp0.01}O_3$ single crystal, for which ultrahigh strain was observed. Original figures from the SEM software with black curves showing the number of counts per second obtained along with the yellow line of 60µm length placed on the crystal surface. Regular runs of these dependencies and coloured maps representing distributions of the Pb, Zr, and Ti ions in the form, respectively, of the bright red, green and blue points on the black background prove good chemical composition of the investigated crystal. The distribution of each element was checked for areas of 600 µm x 700 µm size on the crystal surfaces. The Ti map was magnified to see this ion's regular distribution better.

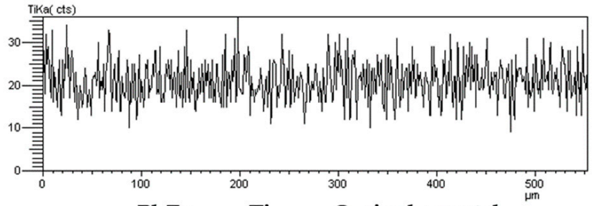

$PbZr_{0.96\pm0.01}Ti_{0.04\mp0.01}O_3$ single crystal

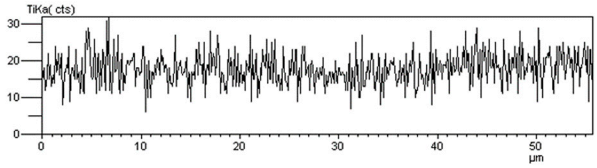

$PbZr_{0.95\pm0.01}Ti_{0.05\mp0.01}O_3$ single crystal

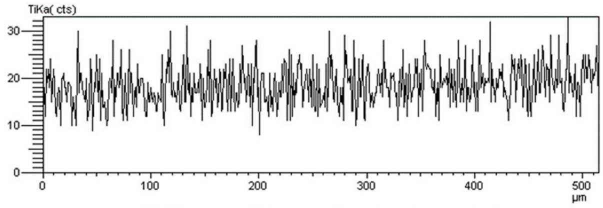

$PbZr_{0.90\pm0.01}Ti_{0.10\mp0.01}O_3$ single crystal

Figure S2-2. Original figures from the SEM software show Ti distribution in PZT single crystals of three different compositions. Lines are counts per second for Ti ion distributed along a line, as in Figure S2-1. Independently on Ti content, these runs are regular and testify about homogeneous chemical compositions of the crystals investigated.
